# A Digitalized Gyroscope System Based on a Modified Adaptive Control Method

**DOI:** 10.3390/s16030321

**Published:** 2016-03-04

**Authors:** Dunzhu Xia, Yiwei Hu, Peizhen Ni

**Affiliations:** Key Laboratory of Micro-inertial Instrument and Advanced Navigation Technology, Ministry of Education, School of Instrument Science and Engineering, Southeast University, Nanjing 210096, China; 220132565@seu.edu.cn (Y.H.); 220132629@seu.edu.cn (P.N.)

**Keywords:** silicon microgyroscope, MEMS, adative control algorithm, rotation mode elimnation, parameter optimization, FPGA

## Abstract

In this work we investigate the possibility of applying the adaptive control algorithm to Micro-Electro-Mechanical System (MEMS) gyroscopes. Through comparing the gyroscope working conditions with the reference model, the adaptive control method can provide online estimation of the key parameters and the proper control strategy for the system. The digital second-order oscillators in the reference model are substituted for two phase locked loops (PLLs) to achieve a more steady amplitude and frequency control. The adaptive law is modified to satisfy the condition of unequal coupling stiffness and coupling damping coefficient. The rotation mode of the gyroscope system is considered in our work and a rotation elimination section is added to the digitalized system. Before implementing the algorithm in the hardware platform, different simulations are conducted to ensure the algorithm can meet the requirement of the angular rate sensor, and some of the key adaptive law coefficients are optimized. The coupling components are detected and suppressed respectively and Lyapunov criterion is applied to prove the stability of the system. The modified adaptive control algorithm is verified in a set of digitalized gyroscope system, the control system is realized in digital domain, with the application of Field Programmable Gate Array (FPGA). Key structure parameters are measured and compared with the estimation results, which validated that the algorithm is feasible in the setup. Extra gyroscopes are used in repeated experiments to prove the commonality of the algorithm.

## 1. Introduction

MEMS gyroscopes are a kind of angular rate sensor widely used in the fields of navigation, motor vehicles, and mobile devices. They provide inertial angular rate measurements with low cost and low power consumption [1,2]. Recent years have witnessed the development of adaptive control methods for MEMS gyroscopes. The application of an adaptive control algorithm can provide on-line estimation of input angular rate and other parameters, thus it has fine robustness features against parameter variances and disturbances.

Park has proposed an adaptive measurement mode for operating MEMS gyroscopes, and formulated a unified methodology for the synthesis and analysis for the algorithm [3]. The algorithm can realize online estimation and compensation of varied defects and disturbances that influence the behavior of a MEMS gyroscope. He also extended the adaptive control method to mode-tuning and angle measurement applications, which can directly measure the rotation angle without integration of the angular rate [4,5]. John presented the concept of an adaptively controlled single-mass tri-axial angular rate (AR) sensor [6]. The single mass is free to move in three directions, thus a tri-axial perfect oscillator model is chosen as a reference. According to the control law, the coefficients of damping, stiffness and input angular rate can be completely estimated. To ensure the parameter convergence in the continuous domain, a modified trajectory algorithm is presented with the aim to reduce the discretization errors. Fei adopted sliding mode control in MEMS gyroscopes [7,8,9]. A proportional and integral sliding surface is defined and applied to calculate the control forces of different axes. In order to eliminate the chattering, the discontinuous control component is replaced by a smoothing sliding mode component. The algorithm is also updated by the robust sliding mode control method and applied on the tri-axial gyroscope model. Leland developed Lyapunov-based adaptive controllers for MEMS gyroscopes to compensate the uncertainty in the natural frequencies, mode coupling and damping. The controllers are tested under averaged low frequency model and full gyroscope model conditions, respectively [10,11]. Dong presented a new adaptive control method based on Active Disturbance Rejection Control (ADRC), which can precisely estimate and compensate the disturbance on each axis, and is applied on a vibrational beam gyroscope [12].

Most of the reported control methods remained at the simulation stage, and others are implemented on analog circuits. Thus, for the first time, we look into the possibilities of realizing the adaptive control algorithm on a digitalized gyroscope system. Once the algorithm is implemented in the digitalized setup, the measured angular rate will not be affected by variance of the coupling coefficients or the quality factor of the gyroscope, thus the system has better adaptivity for different gyroscopes under different conditions.

The algorithm presented in this paper is based on a model reference adaptive control algorithm, and the adaptive control algorithm is further modified based on previous work. The adaptive law is redesigned to estimate the asymmetric coupling parameters and the reference model is replaced with two PLLs for FPGA implementation. Besides, the rotation mode of the gyroscope is considered in this manuscript and the corresponding elimination section is designed. This paper is organized as follows: in Section 2 the structure diagram and the dynamic characteristic of the gyroscope system are first described, and next in Section 3 the adaptive control method with the robust resistance to *z*-axis rotation mode is finally presented. Simulation results and the parameter estimation process are then discussed in Section 4, and finally the algorithm implementation on the FPGA-based digitalized gyroscope system is demonstrated in Section 5.

## 2. Gyroscope System Description

**Figure 1:**
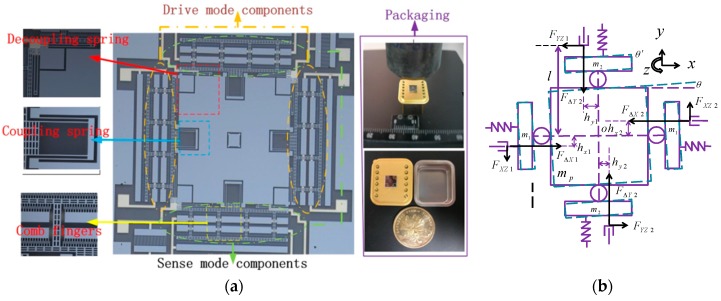
Description of the gyroscope used. (**a**) SEM photo and packaging details of the gyroscope; (**b**) Schematic diagram of the gyroscope rotation mode.

A capacitive silicon micromachined gyroscope is used in this work. The designed *z*-axis gyroscope is a double decoupled single proof mass gyroscope. It is a two-layer structure which mainly consists of the drive mode components, the sense mode components, the proof mass, the damping elements and the glass substrate, *etc*. The details of the gyroscope including its packaging, comb fingers, coupling and decoupling springs are displayed in Figure 1a.

The gyroscope is fabricated by the silicon-on-glass (SOG) process. The upper layer of the gyroscope is the silicon structure ICP etched through the single crystal silicon bulk micromachining, while the lower layer is the glass substrate which is anodically bonded with the silicon structure. The gyroscope used in this work has a resonant frequency of 3195.1 Hz in drive mode and 3105.9 Hz in sense mode. The symmetric structure of the gyroscope is especially suitable for the implementation of the adaptive control algorithm proposed in [4]. The detailed structure parameters of the gyroscope in this work are listed in Table 1.

From the structure model it can be seen that the ideal micromachined gyroscope is a mass-spring-damping system with two independent modes. However, due to the inevitable fabrication errors of the micromachined structure, the actual gyroscope dynamic equation tends to be more complicated. Besides, the fabrication imperfection of comb fingers and beams will contribute to stiffness coupling, stiffness asymmetry, drive force asymmetry and displacement detection asymmetry, *etc.* These structural errors will lead to undesired coupling of drive and sense modes, and even excite other sub-modes, for instance, the differential mode drive force asymmetry will make the proof mass to rotate around z axis. In such case, mechanical model of three freedom degrees system is considered in this paper to study the dynamic characteristic, as illustrated in Equations (1)–(3), and the schematic diagram of the gyroscope rotation mode, is also depicted in Figure 1b.

The drive mode:

(1) $$m_{x}\ddot{x}+D_{xx}\dot{x}+D_{xy}\dot{y}+K_{xx}x+K_{xy}y=F_{dx}+2m_{p}\Omega_{s}\dot{y}$$

The sense mode:

(2) $$m_{y}\ddot{y}+D_{yy}\dot{y}+D_{yx}\dot{x}+K_{yy}y+K_{yx}x=F_{dy}-2m_{p}\Omega_{s}\dot{x}$$

The rotation mode:

(3) $$I_{z}\left(\ddot{\theta }+D_{z}\dot{\theta }+K_{z}\theta \right)=\Sigma F_{\Delta xi}h_{xi}+\Sigma F_{\Delta yi}h_{yi}+\Sigma F_{xzi}l+\Sigma F_{yzi}l+\Sigma F_{xci}h_{xi}+\Sigma F_{yci}h_{yi}\;i=1,2$$

where *x*, *y* is the displacement of the drive axis and sense axis, $\;m_{x}$ and $m_{y}$ is the vibration mass of these two modes, $m_{P}$ is the value of proof mass, $I_{z}$ is the rotary moment of inertia of the proof mass around z axis, and θ is the rotation angle of the z axis. $K_{xx}$, $K_{yy}$ are the normal stiffness coefficients of the drive and sense modes, and $K_{xy}$, $K_{yx}$ are the coupling stiffness coefficients. $D_{xx}$, $D_{yy}$ are the normal damping coefficients of the drive and sense modes, and $D_{xy}$, $D_{yx}$ are the coupling damping coefficients. $F_{dx}$ and $F_{dy}$ are the feedback forces that attached on the drive mode and sense mode, respectively.

As illustrated in Figure 1b, $D_{z}$ and $K_{z}$ are the damping and stiffness coefficient of rotation mode. $F_{\Delta xi}$ and $F_{\Delta yi}$ represent the asymmetric drive forces of the drive and sense axes, $F_{xzi}$ and $F_{yzi}$ are the vibration components that couple from the drive and sense modes to the rotation mode. $F_{xci}$ and $F_{yci}$ are the force that calculated to compensate the asymmetric components, $h_{xi}$ and $h_{yi}$ are the arms of the differential drive forces, i=1,2 indicates the different sides of the electrodes, l is the dimension parameter of the gyroscope, representing the projection on these two axes of the distance from the beam bearing point to the vibration mass centre:

(4) $$F_{xz}=\left(m_{p}+2m_{1}\right)\gamma_{x}\omega_{x}^{2}x$$

(5) $$F_{yz}=\left(m_{p}+2m_{2}\right)\gamma_{y}\omega_{y}^{2}y$$

(6) $$F_{\Delta x}=F_{dx}\lambda_{x}$$

(7) $$F_{\Delta y}=F_{dy}\lambda_{y}$$

where $\gamma_{x}$ and $\gamma_{y}$ represent the rotation mode coupling coefficients of drive and sense modes, $\lambda_{x}$ and $\lambda_{y}$ are the drive force asymmetric coefficients of drive and sense modes. As depicted in Figure 1b, $m_{p}$ is the proof mass, $m_{1}$ and $m_{2}$ are the masses of the fingers and frames that vibrate in the two modes respectively. Thus $m_{p}+2m_{1}$ and $m_{p}+2m_{2}$ are equal to $m_{x}$ and $m_{y}$ in Equations (1) and (2) respectively. $\omega_{x}$ and $\omega_{y}$ are the resonate frequencies of the drive and sense mode respectively. From the equations of the dynamic characteristics of the gyroscope, it can be concluded that the drive and sense modes are coupled with each other, and the *z*-axis mode is influenced by different kinds of disturbances and fabrication imperfections.

Due to the existence of the rotation mode, the measured displacements of drive and sense modes are contaminated by the projection of the rotation movement on the other two modes. Assuming $\hat{x}\;$, $\hat{y}$ to be the displacements of *x* and *y* that influenced by the rotation mode, $\delta_{x}$ and $\delta_{y}$ are the beam fabrication asymmetric coefficients, then we get:

(8) $$\hat{x}=cos\theta x-\delta_{x}lsin\theta$$

(9) $$\hat{y}=cos\theta y+\delta_{y}lsin\theta$$

Considering the control algorithm formation of the drive and sense modes, the gyroscope used in our experiment is double decoupled dual-mode micromachined gyroscope. The drive and sense modes are designed symmetrically, thus the adaptive control algorithm applied on the two modes is also symmetric.

The reference model of the gyroscope is defined as two ideal oscillators, which will remain stable vibration at certain frequencies [4]. The dynamic equations of the ideal reference model can be written as:

(10) $$\ddot{q}+K_{ideal}q=0$$

where the stiffness matrix $K_{ideal}$ is:

(11) $$K_{ideal}=\left[\begin{array}{cc} K_{ideal\_x} & 0\\ 0 & K_{ideal\_y} \end{array}\right]$$

The elements on the leading diagonal are the stiffnesses of the two modes, and the vector q is:

(12) $$q=\left[\begin{array}{c} x\\ y \end{array}\right]$$

Equation (12) represent the displacements of the ideal gyroscope structure.

Considering the coupling terms and the Coriolis force aroused from the input angular rate, the model of the physical gyroscope mechanical structure can be expressed as:

(13) $$\left[\begin{array}{cc} m_{x} & 0\\ 0 & m_{y} \end{array}\right]\left(\ddot{q}+D\cdot \dot{q}+K\cdot q\right)=F_{d}-2m_{p}\Omega \dot{q}$$

In Equation (13) D is the damping matrix, K is the stiffness matrix, and Ω is the matrix constructed from the input angular rate. $F_{d}$ is the vector of the drive force that attached to the drive electrode fingers of the gyroscope.

(14) $$D=\left[\begin{array}{cc} D_{xx} & D_{xy}\\ D_{yx} & D_{yy} \end{array}\right]$$

(15) $$K=\left[\begin{array}{cc} K_{xx} & K_{xy}\\ K_{yx} & K_{yy} \end{array}\right]$$

(16) $$F_{d}=\left[\begin{array}{c} F_{x}\\ F_{y} \end{array}\right]$$

(17) $$\Omega =\left[\begin{array}{cc} 0 & -\Omega_{z}\\ \Omega_{z} & 0 \end{array}\right]$$

Define ***R*** to be the estimation error between the actually stiffness matrix and the reference model:

(18) $$R=K-K_{ideal}=\left[\begin{array}{cc} R_{xx} & R_{xy}\\ R_{yx} & R_{yy} \end{array}\right]$$

Due to the fabrication imperfections, small cross-coupling terms still exist between the two modes. In the matrix, the coupling coefficients $D_{xy}$ and $D_{yx}$, $K_{xy}$ and $K_{yx}$ are not necessarily equal to each other, respectively, thus an algorithm that can deal with each components correspondingly is of great importance.

## 3. The Adaptive Control Algorithm

By comparing the output signal of the reference model and the physical gyroscope, first the tracking error can be obtained. With the proper adaptive law, the estimated parameters are then corrected, thus the parameters are estimated precisely and the feedback control force can be generated through the estimated parameters of the gyroscope, and finally the system is well controlled.

**Figure 2:**
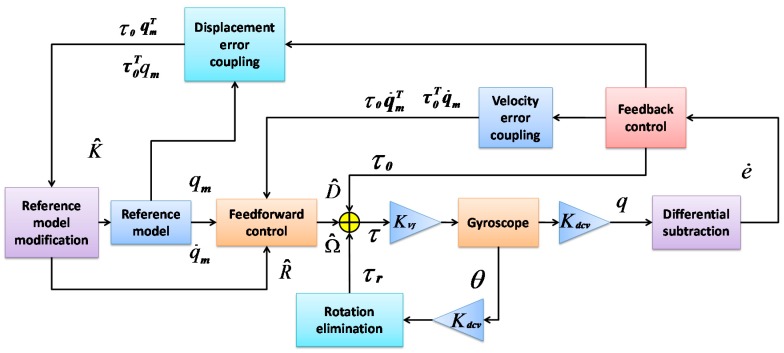
The block diagram of the adaptive control gyroscope system.

The block diagram of the whole system is shown in Figure 2 , where the differential subtract block compares the velocity outputs with the actual gyroscope and the reference model, and generates an velocity error *e*, which contains the information of parameter estimation error:

(19) $$e=q-q_{m}$$

The reference model modification section calculates the estimated stiffness matrix, which is used to control the vibration frequency of the reference model. The feedback control block further generates a direct feedback signal, *i.e.*, the velocity error, which acts as the first feedback signal.

The control law is designed to be implemented in discrete domain. Define the sampling index *k*, and the sampling time Δt, the differential subtraction section provides the estimation error $\Delta e=e_{k+1}-e_{k}$, and multiplied directly by the feedback coefficient $-\gamma_{0},$ *i.e.*,

(20) $$\tau_{0}=-\gamma_{0}\Delta e$$

Meanwhile, the velocity error coupling and displacement error coupling modules are multiplied by the feedback components with the corresponding velocity or displacement signals respectively, then to provide the upgrade amount of different elements in the parameter matrix. In the feedforward control section, the signal that can be used to compensate the coupling and damping disturbances are finally generated, R, D, Ω are modulated with corresponding sinusoidal waves and sent to the two modes.

Assuming $\hat{R},\;\hat{D},\;\hat{\Omega }$ to be the estimated value of matrix R, D, Ω, and $\tilde{R},\;\tilde{D},\;\tilde{\Omega }$ to be the estimation error of the three matrixes, respectively. A positive definite Lyapunov function is chosen as shown in the Appendix B. In the practical conditions, the coupling coefficients on the anti-diagonal of the parameter matrices are not necessarily equal to each other, and the control law will decouple each of the coupling coefficients, respectively. To make the Lyapunov function semi-negative definite, the adaptive control law can be chosen as:

(21) $$\Delta \tilde{R}=\gamma_{R}q_{m}\tau_{0}^{T}$$

(22) $$\Delta \tilde{D}=\gamma_{D}\left(q_{m}\left(k+1\right)-q_{m}\left(k\right)\right)\tau_{0}^{T}$$

(23) $$\Delta \tilde{\Omega }=\gamma_{\Omega }\left(q_{m}\left(k+1\right)-q_{m}\left(k\right)\right)\tau_{0}^{T}$$

The Lyapunov stability analysis of the algorithm is illustrated in the Appendix B. The final output angular velocity signal is derived by integral calculation: $\Omega =\sum^{}\Delta \tilde{\Omega }$ elements on the counter-diagonal of the matrix Ω is the estimated angular velocity.

The reference model modification block estimates the normal stiffness and coupling stiffness of the two gyroscope modes, which will be utilized by the reference model and the feedforward control module.

To maintain the gyroscope working conditions stable, two phase-lock-loop sections are applied in each vibration mode. Each PLL contains a numerical control oscillator (NCO), which generates sinusoidal wave with calculated frequency and phase. Rather than two ideal oscillator models that are illustrated as in Equation (10), the output signal of NCO has a constant amplitude, which will make the algorithm easier for implementation without affecting the Lyapunov stability.

**Figure 3:**
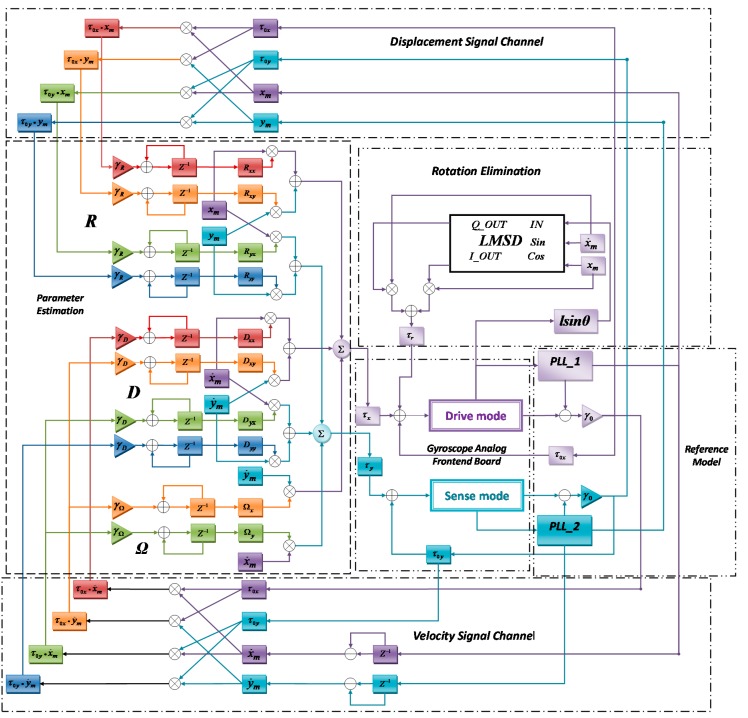
Detailed flow chart of the modified adaptive gyroscope control algorithm.

The detailed flow chart of the adaptive control algorithm of the gyroscope system is shown in Figure 3. With two PLLs acting as the reference model, the control error information can be transferred to estimate different parameters through the displacement signal channel and the velocity signal channel. Additional differential detection section is attached at the drive mode to collect rotation angular information so that the least mean square demodulation (LMSD) section can generate signal $\tau_{r}$ to eliminate such an undesirable rotation.

To fulfill the designed adaptation law, the feedforward control module is designed to update the parameter estimation and generate the final feedforward signal. The final gyroscope angular rate output is also generated from the module. The total drive signal τ is attached on the drive fingers of the two modes. It consists of three parts including the feedforward signal, the feedback signal $\tau_{0}$, and the rotation eliminate signal $\tau_{r}$, *i.e.*,

(24) $$\tau =\hat{D}{\dot{q}}_{m}+\hat{R}q_{m}+2\hat{\Omega }{\dot{q}}_{m}+\tau_{0}+\tau_{r}$$

where $\tau_{r}$ is defined as:

(25) $$\tau_{r}=\left[\begin{array}{c} F_{xc}\\ F_{yc} \end{array}\right]$$

Components of the drive signal have different frequency and phase, in such case the add operation will not severely increase the total drive signal amplitude or make the digital amount overflow. To eliminate the rotation components, the rotation elimination circuit is specially designed. In Equation (3) assume θ=0, and to cancel off the coupling force components in different modes, then we get:

(26) $$F_{xc}=-F_{\Delta x}-F_{xz}l/h_{x}$$

(27) $$F_{yc}=-F_{\Delta y}-F_{yz}l/h_{y}$$

Since the frequencies of the asymmetric drive force and the stiffness coupling components are different, the gyroscope is forced to rotate with the mixture of these two frequencies. The demodulation algorithm is applied in both modes to figure out the rotation components of each frequency and generate the compensation forces $F_{xc}$ and $F_{yc}$ of each frequency. Substituting Equations (6) and (7) into Equations (26) and (27), then we get:

(28) $$F_{xc}=-F_{dx}\lambda_{x}-m_{p}\gamma_{x}\omega_{x}^{2}xl/h_{x}$$

(29) $$F_{yc}=-F_{dy}\lambda_{y}-m_{p}\gamma_{y}\omega_{y}^{2}yl/h_{y}$$

By applying small-angle approximation in Equations (8) and (9), it can be written as:

(30) $$\hat{x}=x-h_{x}\theta$$

(31) $$\hat{y}=y+h_{y}\theta$$

The unmatched signals of the drive modes and sense modes are measured from the gyroscope detection pins by signal condition circuits and calculated in the FPGA chip. Although the frequencies of the signals are predictable, the phases of the detected signals remain unknown. Due to the fabrication variation, the coefficients of the gyroscope structure are not measureable, thus a demodulation method is applied to estimate the amplitude of the signal. Here the LMSD algorithm is applied in both modes. The demodulation reference signals are provided by the reference model, the calculated results are multiplied with a pair of quadrature reference signals to generate the appropriate rotation elimination signal $\tau_{r}$.

The rotation angle θ contains the frequency components of both modes oscillation frequencies. From the detection output port of each mode, the differential signal is demodulated with a pair of quadrature sinusoidal or cosine signals of a certain frequency. Assuming the detected rotation signal is lsinθ, it contains different frequency components of ω and (ω+Δω), with the corresponding magnitude of A, B, C, E, respectively. By applying the LMSD algorithm, the rotation signal can be easily decomposed and recombined with two quadrature reference sinusoidal signals:

(32) lsinθ=Asin(ωt)+Bcos(ωt)+Csin(ωt+Δωt)+Ecos(ωt+Δωt)=(A+CcosΔωt−EsinΔωt)sin(ωt)+(B+Csinφt+EcosΔωt)cos(ωt)

**Figure 4:**
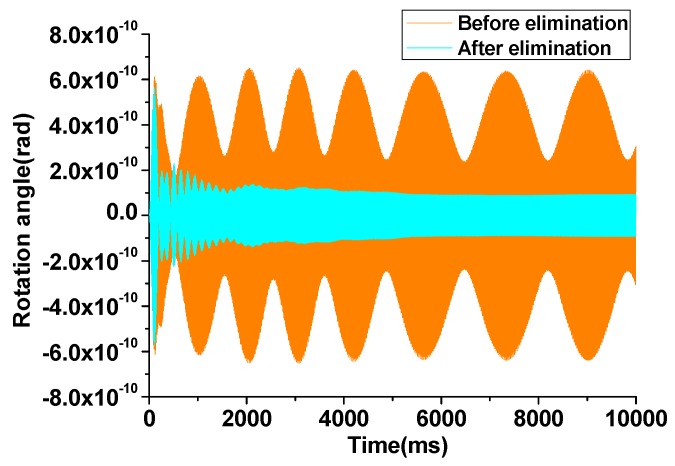
Rotation angular output before and after the elimination circuit.

**Figure 5:**
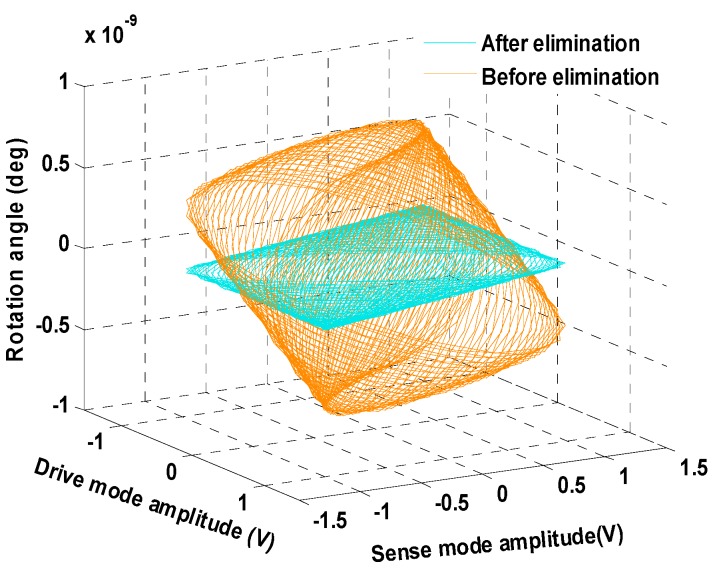
The Lissajous trajectories plotted with the output of three modes.

From Equation (32) it can be seen that, although the original frequency components cannot be restored, the force components that are acquired to eliminate the rotation are calculated in real time and can be attached to the drive fingers of the corresponding mode. With the utilization of the rotation elimination circuit, the unexpected rotation of the proof mass can be effectively suppressed to be less than $2\times 10^{-10}$ rad, compared with the former angular output of $6\times 10^{-10}$ rad. The rotation angular output and the Lissajous trajectories for tri-modes are depicted in Figure 4 and Figure 5, respectively.

From the simulation result illustrated in Figure 4 and Figure 5, it can be concluded that the algorithm based on the LMSD can effectively suppress the rotation mode of the gyroscope. With the differential movement of exited by the rotation is suppressed, the gyroscope works at a more stable condition and the control and measurement precision is also enhanced.

In terms of different modes displacement detection, the differential mode signals are extracted in the drive and sense modes, while the common mode signal are picked out in the rotation mode. In this case, the asymmetric displacement of drive and sense modes are only generated by the asymmetric drive force, *i.e.*, their rotation angle and linear displacement signals are totally decoupled. Therefore, the rotation elimination circuits are relatively independent of the adaptive control circuits for the drive and sense modes.

Theoretically, the Lyapunov stability of the adaptive algorithm controlled system is not affected by rotation section. The main parameters and coefficients of the simulated system are listed as in Table 2. In the implementation process of the algorithm, apart from the coefficients of the mechanical-electrical interface and the parameter of the gyroscope dynamical model, all the variables and coefficients are calculated or stored in the FPGA chip.

Compared with the conventional adaptive control algorithm proposed in [3], the modified adaptive control algorithm proposed in this work has the following improvements: the adaptive law is redesigned to suit the asymmetric condition; rotation elimination section is added to suppress the differential components in the detection signal; the reference model is replaced with two NCOs and the modified adaptive law has a simpler arithmetic structure, the algorithm is more suitable for FPGA implementation.

## 4. Algorithm Simulation

Simulation of the adaptive control based gyroscope system consists of two steps. The continuous domain simulation verifies the adaptive control algorithm and the digital domain simulation based on the DSP builder tool provides convenient conversion from control algorithm simulation to hardware program language realization.

Simulation system is built in Simulink to investigate the performance of the proposed algorithm. Changing of the gyroscope parameters is simulated to verify the adaptive characteristic of the algorithm. The influences of the coefficients on the gyroscope system performance are also compared in this section.

**Figure 6:**
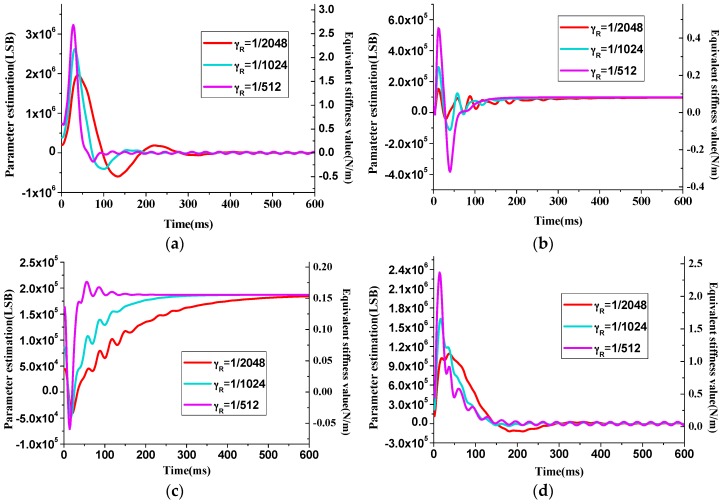
Convergence process curves of stiffness estimation error matrix **R** with different $\gamma_{R}$ values. (**a**) $\;R_{xx}$, (**b**) $R_{xy}$, (**c**) $R_{yx}$, (**d**) $R_{yy}$.

**Figure 7:**
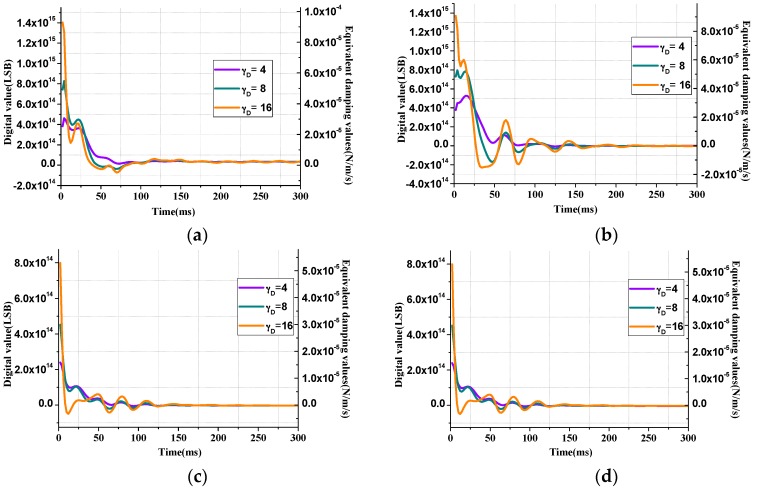
Convergence process curves of damping estimation matrix ***D*** with different $\gamma_{D}$ values. (**a**) $D_{xx}$, (**b**) $D_{xy}$, (**c**) $D_{yx}$, (**d**) $D_{yy}$.

The value of $\gamma_{R}$ and $\gamma_{D}$ will also affect the estimation convergence speed of **R** and **D** matrix, respectively, as shown in Figure 6 and Figure 7. From the curves that represent estimation processes with different $\gamma_{R}$ values, it can be inferred that larger $\gamma_{R}$ can provide faster stiffness estimation but has larger overshoot and may result in overflow of the digital output signal.

Like the case of $\gamma_{R}$ value, $\gamma_{D}$ also has the influences on the overshoot and estimation time. Moreover, the estimation result of damping components are multiplied by the velocity of the reference model, in such case, the system vibration condition is sensitive to the signal. When $\gamma_{R}$ value is too large, such as the orange curve in Figure 7d, ripples will emerge in the parameter estimation result, and the amplitude control process is disturbed.

**Figure 8:**
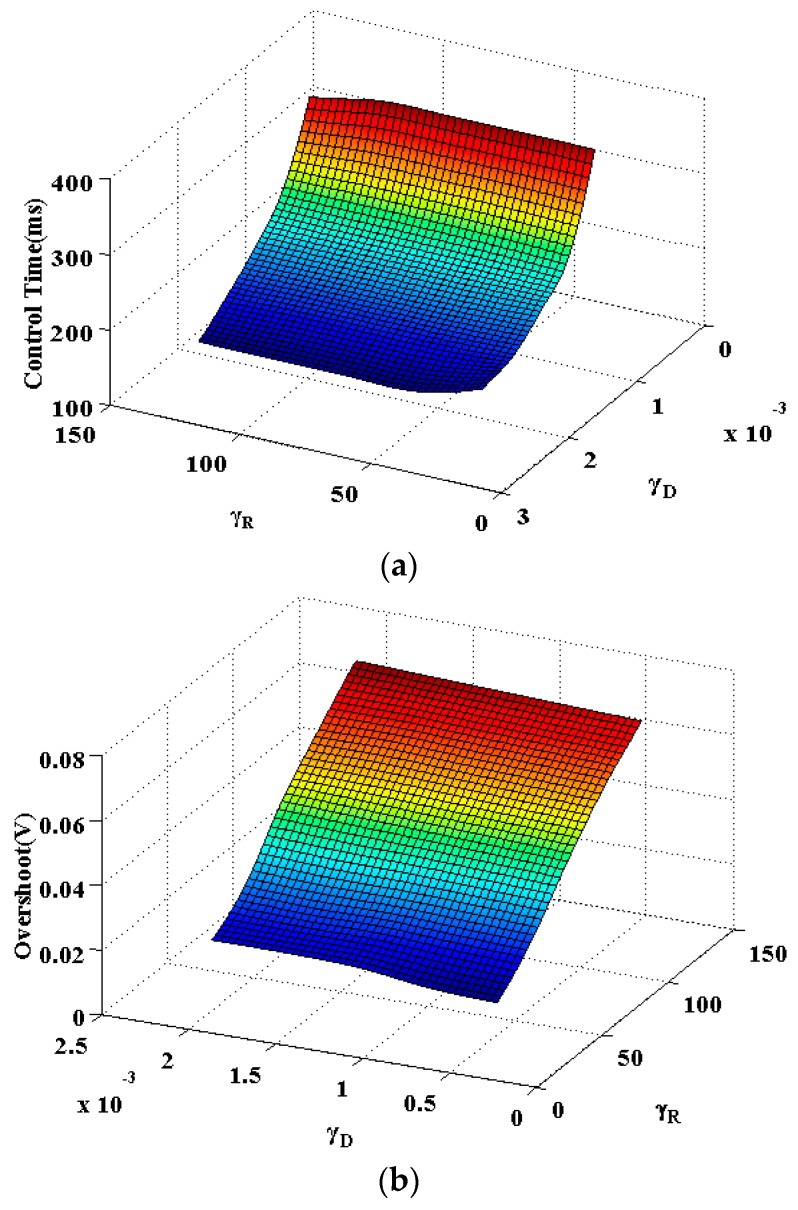
The amplitude control performance with different $\gamma_{R}$ and $\gamma_{D}$ values. (**a**) Control time, (**b**) Overshoot.

From Figure 8 it can be concluded that $\gamma_{D}$ will influence the maximum overshoot during the start-up period and $\gamma_{R}$ value mainly relates with the time consumption of the amplitude control. The adaptive algorithm coefficients$\omega_{R}$, $\gamma_{D}$ will influence the amplitude control speed of the start-up process, thus a proper combination of these two values is necessary. After a trade-off is made between the control time, overshoot and the proper estimation of parameters that illustrated in Figure 6 and Figure 7, the coefficients are finally determined with a multi-object optimization function [12,13].

**Figure 9:**
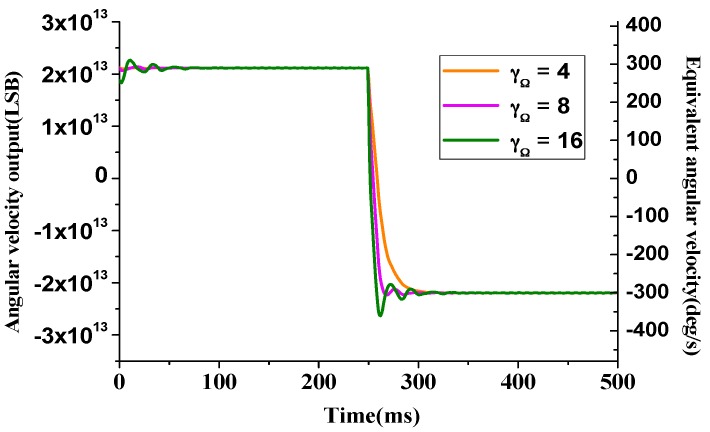
Angular velocity output with step-formed input signal.

To further investigate the speed performance of input angular rate estimation, the angular rate estimation function is tested with the ±300°/s step-formed input signal. The angular rate estimation parameter can affect the slew rate and overshoot of the angular rate, as depicted in Figure 9.

**Figure 10:**
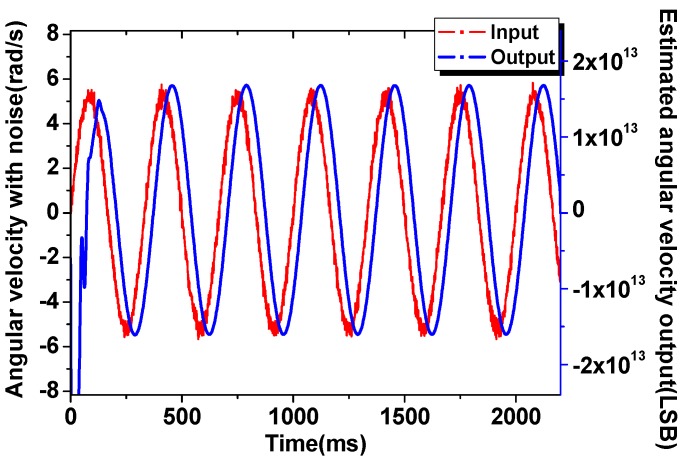
Noise suppression of the input angular rate estimation.

With the integral effect, the adaptive control method also has a fine performance against the input noise, from Figure 10 it can be calculated that, the white noise mixed with the input signal are effectively suppressed to obtain the clean output angular rate estimation signal, and specifically the SNR (Signal to noise ratio) has been greatly enhanced from 49 dB to 90 dB.

In the other hand, $\gamma_{\Omega }$ is also simultaneously related with the measurement precision and bandwidth of the gyroscope system. These two characteristics are contradict against each other, thus a trade-off should be made according to the expected application of the gyroscope system.

**Figure 11:**
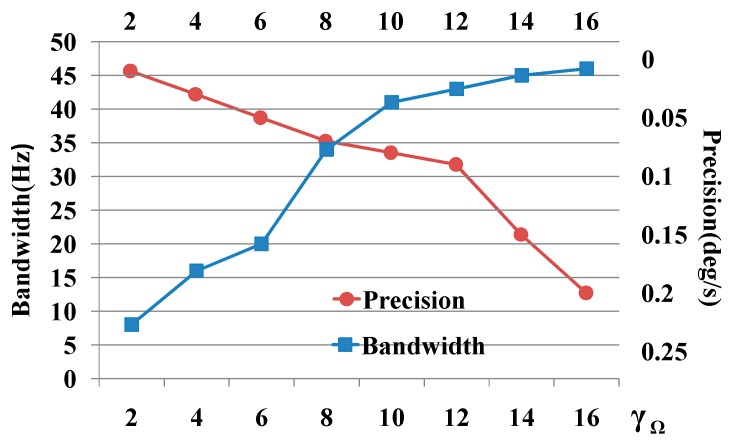
Measurement precision and bandwidth with different $\gamma_{\Omega }$ values.

As shown in Figure 11, to meet the gyroscope application requirement, the bandwidth should be more than 40 Hz, and the measurement precision should be higher than 0.1°/s, finally we choose the $\gamma_{\Omega }$ value as 12.

**Figure 12:**
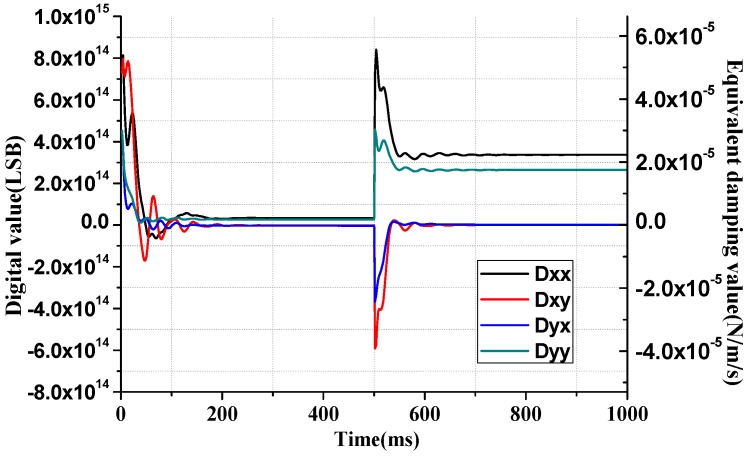
Damping coefficient estimation with switch change at *t* = 0.5.

**Figure 13:**
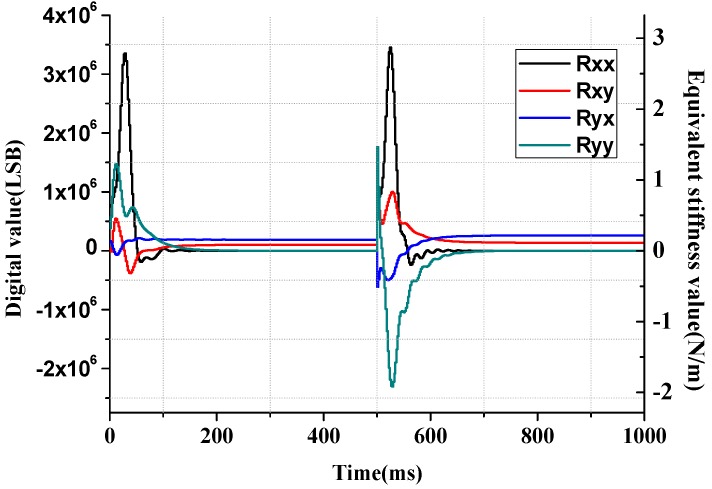
Stiffness coefficient estimation with switch change at *t* = 0.5 s.

With the coefficients of the algorithms are determined, the performance against the parameters variation are also simulated. In practical circumstances, the stiffness of working gyroscope may change due to the environmental factors, especially the temperature. In practical condition, the parameters are changed gradually, but to test the estimation speed of the algorithm, the variations are simulated in the form of step switches, as shown in Figure 12 and Figure 13.

In Figure 12, the quality factors of the gyroscope model vibration modes are changed to 1/10 of their original value, thus the damping coefficients are changed accordingly. In Figure 13, the resonating frequencies of the drive mode and the sense mode are both changed by 10 Hz at 500 ms, *i.e.*, from 3715 Hz to 3725 Hz in drive mode and from 3684 Hz to 3674 Hz in sense mode.

From the simulation curves it can be seen that the algorithm can adapt to the parameter variations robustly and the estimated results converge to the real values after a short transition period.

## 5. Experimental Validation

Based on a set of digitalized gyroscope system, the modified adaptive control algorithm is programmed in the FPGA chip to control the gyroscope.

### 5.1. Open-Loop Measurement of the Parameters

To validate the estimation performance of the algorithm, the gyroscope parameters are measured in open-loop testing in advance [14]. As illustrated in Section 2, parameters of the gyroscope are contained in the three modes that coupled with each other. Through frequency sweep and half-power measurement, the resonance frequency $\omega_{0}$ and quality factor Q can be derived, along with the stiffness and damping coefficient of each mode, *i.e.*, $\;K_{xx}$, $K_{yy},\;D_{xx}$ and $D_{yy}$. The coupling coefficients can be calculated according to the mode-coupling transfer function and the gyroscope coupling output. However, the mechanical and electrical coefficients cannot be measured directly, and thus the drive mode force-voltage transfer functions are not available. To solve this dilemma, the force-displacement transfer functions are transformed into voltage-voltage transfer function and measurement of the unknown coefficients are avoided.

The scale factor ratio of drive and sense modes is measured through exiting the rotation mode of the gyroscope when the common-mode force is attached at the drive mode. Assuming $F_{x\;}\left(s\right)$ and $F_{y}\left(s\right)$ to be the resultant forces attached on the drive mode and sense mode, the dynamics equation of the drive and sense modes can be expressed as:

(33) $$\frac{x\left(s\right)}{F_{x}\left(s\right)}=\frac{1}{m\left(s^{2}+\omega_{x}/Q_{x}\cdot s+\omega_{x}^{2}\right)}$$

(34) $$\frac{y\left(s\right)}{F_{y}\left(s\right)}=\frac{1}{m\left(s^{2}+\omega_{y}/Q_{y}\cdot s+\omega_{y}^{2}\right)}$$

and under such drive force, the displacement of sense mode can be described as:

(35) $$y\left(s\right)s^{2}+c_{yy}y\left(s\right)s+y\left(s\right)=y\left(s\right)\cdot H_{y}\left(s\right)=K_{xy}x\left(s\right)+D_{xy}\cdot s\cdot x\left(s\right)$$

where:

(36) $$H_{y}\left(s\right)=\frac{1}{m\left(s^{2}+\frac{\omega_{y}}{Q_{y}}\cdot s+\omega_{y}^{2}\right)}$$

Considering the scale factor ratio of drive and sense modes *p*, Equation (36) can be written as:

(37) $$\frac{y\left(s\right)}{x\left(s\right)}=p\cdot \frac{U_{y}\left(s\right)}{U_{x}\left(s\right)}=K_{xy}\cdot H_{y}\left(s\right)+D_{xy}\cdot s\cdot H_{y}\left(s\right)$$

By frequency sweeping around the resonating frequency, the coupling coefficients can be derived from least square method. The detailed procedure is described as:

(38) $$\frac{y\left(s\right)}{x\left(s\right)}=m+nj$$

(39) $$H_{y}\left(s\right)=a+bj$$

(40) $$s\cdot H_{y}\left(s\right)=c+dj$$

where *m, n* are measured data, and *a, b, c, d* can be calculated through the gyroscope transfer function.

Assuming the frequency sweeping points are $\omega_{1},\omega_{2}\ldots \omega_{n}$, the corresponding identification equations can be written in matrix format that listed as in Equation (41).

(41) $$\left[\begin{array}{c} \begin{array}{c} m\left(\omega_{1}\right)\\ m\left(\omega_{2}\right) \end{array}\\ \vdots \\ m\left(\omega_{n}\right) \end{array}\right]=\left[\begin{array}{c} \begin{array}{cc} a\left(\omega_{1}\right) & c\left(\omega_{1}\right)\\ a\left(\omega_{2}\right) & c\left(\omega_{2}\right) \end{array}\\ \begin{array}{cc} \vdots & \vdots \\ a\left(\omega_{n}\right) & c\left(\omega_{n}\right) \end{array} \end{array}\right]\cdot \left[\begin{array}{c} K_{xy}\\ D_{xy} \end{array}\right]$$

Equation (41) can be written in the form of:

(42) $$M_{n\times 1}=A_{n\times 2}\cdot P_{2\times 1}$$

By operating least square procedure, the calculated parameters are obtained under the meaning of least variance, *i.e.*:

(43) $$\tilde{P}={\left(A\prime \cdot A\right)}^{-1}\cdot A\prime \cdot M$$

**Figure 14:**
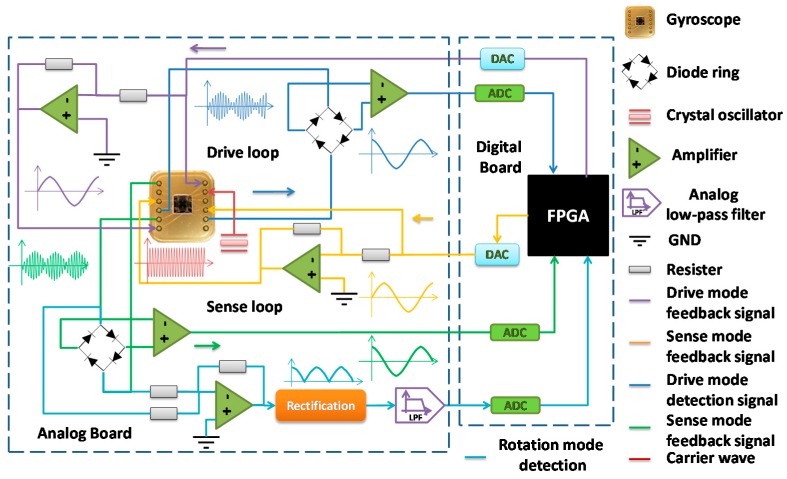
Schematic diagram of the gyroscope system circuit.

To measure the coupling coefficients that form the sense mode to the drive mode, the open-loop drive signal is attached on the sense mode and the coupling signal is measured from drive mode. Conducting the same process as illustrated above, $K_{yx}$ and $D_{yx}$ can be calculated. The schematic diagram of the gyroscope system circuit is shown in Figure 14, when used in the open-loop measurement, the digital board is removed and external signals are attached at the drive electrodes of the two modes. A crystal oscillator is used to provide carrier wave of the gyroscope, and capacitances variance signals of the two modes are detected by two diode rings. Since double-sided drive method is applied in the gyroscope, feedback signals are transferred through inverting amplifiers and attached on the opposite drive electrodes.

### 5.2. Experimental Implementation Result and Comparison

For the convenience of the algorithm implementation, the simulation is performed with DSP builder toolbox, where algorithms of the system are verified and the word length and truncation of each section are determined. Most of the sections in the algorithm can be resolved into basic addition, subtraction, multiplication and integral calculations. The most complex and resource consumption section is the reference model with two NCOs, which are implemented using CORDIC algorithm [15]. As for the calculation of the matrixes, different elements are calculated and transferred separately through different branches. Since the usage of fixed point multiplier will consume large amount of hardware resources in the FPGA and add the power consumption of the digitalized system, most of the constant coefficients are determined as powers of 2, thus the multiplication operation can be realized by the shifting operation.

**Figure 15:**
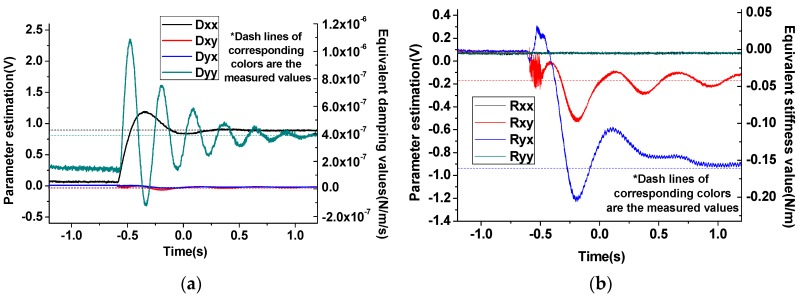
Experimental parameter estimation. (**a**) Damping values; (**b**) Stiffness values.

After the algorithm implementation, values of the parameters estimated program are output from DA chips and recorded, as plotted in Figure 15. In the plots, right vertical axes are labeled with the physical amount equivalent to the digital value system. The dash lines marked with the corresponding curves are the measured values acquired from the open-loop experiment.

By comparing the measurement result with the estimated value, the performance of the adaptive algorithm can be evaluated. Parameters Estimated in the program and measured from open-loop method are compared in Table 3. In Figure 15 the measured values are marked with the dash lines in corresponding colors. From Figure 15 and Table 3 it can be concluded that the estimation results of the adaptive control algorithm highly consistent with the open-loop measurement results in the primary values, and less estimation precision in the weak coupling coefficients that has slight influence in the control system, like $D_{xy}\text{ and }D_{yx}$.

**Figure 16:**
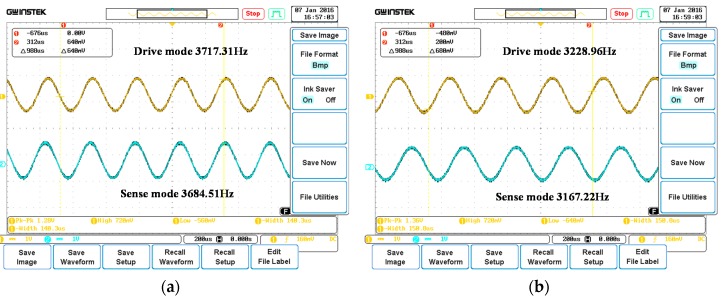
Drive and sense mode signal when controlled by the adaptive algorithm. (**a**) Gyroscope 2; (**b**) Gyroscope 3.

To validate the commonality of the algorithm, another two gyroscopes in the same batch are also picked for repeated test using the same digitalized circuit. Parameters of the corresponding gyroscopes are listed in Table 4, and a screenshot of the vibration of the two modes is shown in Figure 16.

**Figure 17:**
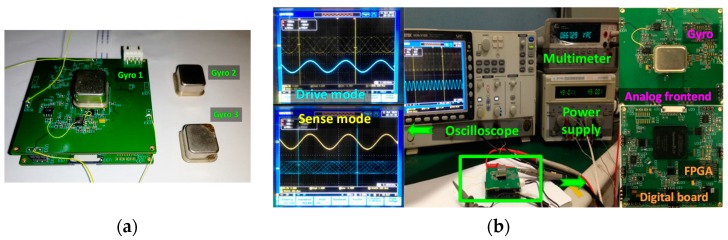
The modified adaptive control system. (**a**) Digitalized system with three gyroscopes used; (**b**) Experimental setup.

The experimental setup established in our work is presented as in Figure 17. The gyroscope system consists of an analog front end, a gyroscope and a digital board based on an FPGA chip. The program is down loaded into an EPCS16 flash chip, so that once the system is supplied with a constant voltage of ±5 V, the algorithm will control the two modes vibrate at constant frequency and amplitude. As can be measured from the oscilloscope, the drive mode frequency is 3105.9 Hz and the sense mode frequency is 3195.1 Hz.

**Figure 18:**
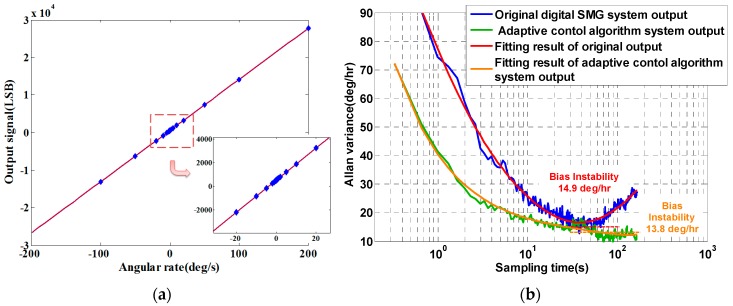
Performance of the system. (**a**) Scale factor; (**b**) Allan variance compared with original system.

As shown in Figure 18, the gyroscope system has scale factor of 136 LSB/°/s, with a nonlinearity of 339 ppm. According to the Allan variance result, bias instability is 13.8°/h, better than 14.9°/h of the digitalized system without adaptive control algorithm [14]. Compared with the output result of the same gyroscope with conventional control algorithm, the improvement of Allan variance can be attributed to the stability of the reference model. The FPGA utilized in our work is Cyclone EP4CE40, and a total of 8125 logic elements (LE) are consumed when the whole modified adaptive control algorithm and AD/DA convertor interface sections are programmed. It can be concluded from the experimental results that the digitalized adaptive control algorithm is successfully applied on the FPGA chip and achieve fine performance. Further enhancement of the performance will be carried out in our future research.

## 6. Conclusions

In this paper we present a digitalized micromachined gyroscope based on a modified adaptive control method. The algorithm can estimate some of the gyroscope key coefficients in real time and make both the drive and sense modes vibrate at constant amplitude, which will enhance the system robustness against environmental variances and disturbances. The rotation mode is also considered in the model, with the corresponding parameters estimated and the common mode vibration amplitude is well controlled, the differential movement exited by the rotation mode is also investigated. Differential detection and LMS demodulation methods are used in the system to eliminate such rotation. Performance including the parameter estimation and the system dynamic characteristics are investigated to choose a set of optimized key coefficients. To validate the commonality of the algorithm, another two gyroscopes are used for repeated test. The digitalized gyroscope system applied with the adaptive control method achieves scale factor of 136 LSB/°/s, with a nonlinearity of 339 ppm, and bias instability of 13.8 °/h by Allan variance.

Comparison of various adaptive control methods are summarized in Table 5. In recent years, different adaptive control methods have been tried on different gyroscopes by some leading researchers. Due to the algorithm complexity, some works still focused on the model building, adaptive control algorithm deduction and simulation verification. Normally, the adopted gyroscope should be designed symmetrically, where *z*-axis MEMS gyroscope and tri-axial gyroscope can be always utilized. Based on the previous work, the modified adaptive control method is experimentally verified and realized on our digital gyroscope system, which further proves that the adaptive control method is feasible in real application.

Future research of the adaptive method controlled gyroscope system may focus on the further enhancement of the performance and application of the adaptive control method on other types of gyroscopes for instance, ring, disk or hemispherical shell gyroscopes.

## Figures and Tables

**Table 1:** The structure parameters of the gyroscope.

Parameter	Value (unit)
Structure thickness	65 (μm)
Proof mass size	2500 × 2500 (μm^2^)
Equivalent drive/sense mass	1.39 × 10^−6^ (kg)
U-shaped spring width	10 (μm)
Drive/sense U-shaped spring length	520 (μm)
Drive/sense beam size	2500 × 70 (μm^2^)
Comb finger width	4 (μm)
Comb finger length	40 (μm)
Comb finger gap	4 (μm)
Comb finger overlap length	20 (μm)
Drive/sense frequency	3194/3105.6 (Hz)
Q-factor (drive/sense mode)	8256/7839

**Table 2:** Coefficients and parameters of the simulated system.

Block	Parameter/Coefficient	Symbol	Continuous Domain Value	Digital Domain Value
Feedforward control	Angular rate integral coefficient	$\gamma_{\Omega }$	0.0087	8
	Damping integral coefficient	$\gamma_{D}$	0.0021	8
	Stiffness error integral coefficient	$\gamma_{R}$	139.5	1/1024
Reference model	Feedback gain	$\gamma_{0}$	$1.9\times 10^{-6}$	16
Mechanical-electricalInterface	Analog-digital domain gain	$K_{ad}$	$2.07\times 10^{5}\;\text{LSB}/V$	-
	Digital-analog domain gain	$K_{da}$	$4.76\times 10^{-6}\;V/\text{LSB}$	-
	Voltage-force coefficient	$K_{vf}$	$2.8\times 10^{-7}\;N/V$	-
	Displacement-capacitance-voltage coefficient	$K_{dcv}$	$4.4137\times 10^{5}\;V/m$	-
Gyroscope dynamical model	Gyroscope proof mass	m	$1.39\times 10^{-6}\;kg$	-
	Drive mode quality factor	$Q_{x}$	8256	-
	Sense mode quality factor	$Q_{y}$	7839	-
	Drive mode resonating frequency	$\omega_{x}$	3194 Hz×2π	-
	Sense mode resonating frequency	$\omega_{y}$	3105.6 Hz×2π	-
	Sense-drive stiffness coefficient	$K_{xy}$	0.1557 N/m	-
	Drive-sense stiffness coefficient	$K_{yx}$	0.1514 N/m	-
	Sense-drive damping coefficient	$D_{xy}$	$1.08\times 10^{-7}\;N/m/s$	-
	Drive-sense damping coefficient	$D_{yx}$	$0.9\times 10^{-7}\;N/m/s$	-

**Table 3:** Comparison between the measurement value and the estimated value.

Parameter	Symbol	Measured Value (Open-Loop)	Estimated Value (Modified Adaptive Control)
Drive mode normal stiffness coefficient	$K_{xx}$	559.3 N/m	559.7 N/m
Stiffness coupling coefficient	$K_{xy}$	0.03 N/m	0.04 N/m
	$K_{yx}$	0.155 N/m	0.17 N/m
Sense mode normal stiffness coefficient	$K_{yy}$	528.8 N/m	528.9 N/m
Drive mode normal damping coefficient	$D_{xx}$	$4.10\times 10^{-6}\;N/m/s$	$4.4\times 10^{-6}\;N/m/s$
Damping coupling coefficient	$D_{xy}$	$2\times 10^{-7}\;N/m/s$	$1\times 10^{-7}\;N/m/s$
	$D_{yx}$	$1\times 10^{-7}\;N/m/s$	$8\times 10^{-8}\;N/m/s$
Sense mode normal damping coefficient	$D_{yy}$	$3.92\times 10^{-6}\;N/m/s$	$4.1\times 10^{-6}\;N/m/s$

**Table 4:** Parameters of gyroscope 2 and gyroscope 3.

Parameter	Symbol	Gyroscope 2	Gyroscope 3
Drive mode normal stiffness coefficient	$K_{xx}$	756.6 N/m	570.6 N/m
Stiffness coupling coefficient	$K_{xy}$	$1.5\times 10^{-3}\;N/m$	$1.13\times 10^{-3}\;N/m$
	$K_{yx}$	$1\times 10^{-3}\;N/m$	$7.4\times 10^{-4}\;N/m$
Sense mode normal stiffness coefficient	$K_{yy}$	744.1 N/m	549.7 N/m
Drive mode resonating frequency	$f_{x}$	3717.31 Hz	3228.96 Hz
Drive mode quality factor	$Q_{x}$	10297	3487
Drive mode normal damping coefficient	$D_{xx}$	$4.48\times 10^{-6}\;N/m/s$	$1.15\times 10^{-5}\;N/m/s$
Damping coupling coefficient	$D_{xy}$	$1\times 10^{-8}\;N/m/s$	$7.9\times 10^{-8}\;N/m/s$
	$D_{yx}$	$6\times 10^{-9}\;N/m/s$	$7.1\times 10^{-8}\;N/m/s$
Sense mode normal damping coefficient	$D_{yy}$	$6.18\times 10^{-6}\;N/m/s$	$1.18\times 10^{-5}\;N/m/s$
Sense mode resonating frequency	$f_{y}$	3684.51 Hz	3167.22 Hz
Sense mode quality factor	$Q_{y}$	8986	4055

**Table 5:** Comparison of different gyroscope control methods in recent years.

Institute	Year	Control Method	Gyroscope Used	Simulation and Experiment
University of California at Berkeley [3]	2000	Adaptive control strategy	*z*-axis MEMS gyroscope	Algorithm simulation
RMIT University [6]	2006	Tri-axial adaptively controlled algorithm	Single-mass tri-axial AR sensor	Modeled fabrication and algorithm simulation
University of Louisiana at Lafayette [7]	2009	Sliding mode control	*z*-axis MEMS gyroscope	Algorithm simulation
Cleveland State University [12]	2008	Active Disturbance Rejection Control	Vibrational beam gyroscope	Analog circuit implementation
This work	2015	Modified Adaptive control	*z*-axis MEMS gyroscope	Simulation and FPGA digital circuit implementation

## Appendix A

### Nomenclature

**Symbol**	**Nomenclature**	**Symbol**	**Nomenclature**
$K_{ideal}$	Ideal Stiffness matrix	$\tau_{0}$	Feedback section output vector
θ	*Z* axis rotation angle	τ ****	Total drive signal matrix
q	Displacement vector	$\tau_{r}$	Rotation elimination signal vector
x	Drive mode displacement	$m_{p}$	Gyroscope proof mass
y	Sense mode displacement	$m_{x}$	Drive mode vibration mass
F	Feedback force matrix	$m_{y}$	Sense mode vibration mass
$F_{x}$	Drive mode feedback force	$Q_{x}$	Drive mode quality factor
$F_{y}$	Sense mode feedback force	$Q_{y}$	Sense mode quality factor
D	Damping matrix	$\gamma_{\Omega }$	Angular rate integral coefficient
K	Stiffness matrix	$\gamma_{D}$	Damping integral coefficient
R	Stiffness estimation error matrix	$\gamma_{R}$	Stiffness error integral coefficient
Ω	Angular rate matrix	$\gamma_{0}$	Feedback gain
e	Displacement estimation error vector	V	Lyapunov function

## Appendix B

The Lyapunov stability proof process is illustrated as follows:

(B1) $$V=\frac{1}{2}\left(\Delta e^{T}\gamma_{0}\Delta e+e^{T}\gamma_{0}Ke+tr\left\{\gamma_{R}^{-1}\tilde{R}{\tilde{R}}^{T}+\gamma_{D}^{-1}\tilde{D}{\tilde{D}}^{T}+\gamma_{\Omega }^{-1}\tilde{\Omega }{\tilde{\Omega }}^{T}\right\}\right)$$

By taking differential operation on the Lyapunov function, it can be derived that:

(B2) $$\Delta V=V_{k+1}-V_{k}=\gamma_{0}\Delta e^{T}\left(\Delta e_{k+1}-\Delta e_{k}\right)+\gamma_{0}{\left(\Delta e_{k+1}-\Delta e_{k}\right)}^{T}K_{m}e+tr\left\{\gamma_{R}^{-1}{\tilde{R}}_{k}\Delta {\tilde{R}}^{T}+\gamma_{D}^{-1}{\tilde{D}}_{k}\Delta {\tilde{D}}^{T}+\gamma_{\Omega }^{-1}{\tilde{\Omega }}_{k}\Delta {\tilde{\Omega }}^{T}\right\}$$

(B3) $$\Delta \tilde{R}={\tilde{R}}_{k+1}-{\tilde{R}}_{k}$$

(B4) $$\Delta \tilde{D}={\tilde{D}}_{k+1}-{\tilde{D}}_{k}$$

(B5) $$\Delta \tilde{\Omega }={\tilde{\Omega }}_{k+1}-{\tilde{\Omega }}_{k}$$

Assuming the gyroscope controlled by the modified control law is under a stable condition, and the control signal is composed of the feedback and feed forward outputs, *i.e.*:

(B6) $$\ddot{q}+D\dot{q}+Kq=\hat{D}{\dot{q}}_{m}+\hat{R}q_{m}+2\hat{\Omega }{\dot{q}}_{m}-\gamma_{0}\dot{e}-2\Omega \dot{q}$$

(B7) $$\ddot{e}+K_{m}e=\hat{D}{\dot{q}}_{m}-D\dot{q}+\hat{R}q_{m}-Rq+2\hat{\Omega }{\dot{q}}_{m}-2\Omega \dot{q}-\gamma_{0}\dot{e}\;=\hat{D}{\dot{q}}_{m}-D\dot{q}+\hat{R}q_{m}-Rq+2\hat{\Omega }{\dot{q}}_{m}-2\Omega \dot{q}-\gamma_{0}\dot{e}\;=\tilde{D}{\dot{q}}_{m}+\tilde{R}q_{m}+2\tilde{\Omega }{\dot{q}}_{m}-\gamma_{0}\dot{e}$$

After left multiplication by $\gamma_{0}\Delta e^{T}$ on both sides of Equation (B7), and written in discrete time domain, it can be derived that:

(B8) $$\begin{array}{c} \gamma_{0}\Delta e^{T}\left(\Delta e_{k+1}-\Delta e_{k}\right)+\gamma_{0}\Delta e^{T}K_{m}e\\ =\gamma_{0}\Delta e^{T}\left(\tilde{D}\left(q\left(k+1\right)-q\left(k\right)\right)+\tilde{R}q\left(k\right)+2\tilde{\Omega }(q\left(k+1\right)-q\left(k\right)\right)-\gamma_{0}^{2}\Delta e^{T}\Delta e \end{array}$$

Substituting Equation (B8) into Equation (B2), the differential Lyapunov function can be written as:

(B9) $$\Delta V=\gamma_{0}\Delta e^{T}\left(\Delta e_{k+1}-\Delta e_{k}\right)+\gamma_{0}\Delta e^{T}K_{m}e+tr\left\{\gamma_{R}^{-1}{\tilde{R}}_{k}\Delta {\tilde{R}}^{T}+\gamma_{D}^{-1}{\tilde{D}}_{k}\Delta {\tilde{D}}^{T}+\gamma_{\Omega }^{-1}{\tilde{\Omega }}_{k}\Delta {\tilde{\Omega }}^{T}\right\}\;=-\tau_{0}\left(\left(\tilde{D}\left(q_{m}\left(k+1\right)-q_{m}\left(k\right)\right)+\tilde{R}q_{m}\left(k\right)+2\tilde{\Omega }(q_{m}\left(k+1\right)-q_{m}\left(k\right)\right)\right)\;-\gamma_{0}^{2}\Delta e^{T}\Delta e+tr\left\{\gamma_{R}^{-1}{\tilde{R}}_{k}\Delta {\tilde{R}}^{T}+\gamma_{D}^{-1}{\tilde{D}}_{k}\Delta {\tilde{D}}^{T}+\gamma_{\Omega }^{-1}{\tilde{\Omega }}_{k}\Delta {\tilde{\Omega }}^{T}\right\}$$

To judge whether Equation (B9) is negative semi-definite, the first term should be combined with the third term and convert into the formula of matrix trace, taking $\tau_{0}\tilde{R}q$ as example:

(B10) $$\tau_{0}\tilde{R}q_{m}=\left[\begin{array}{cc} \tau_{x} & \tau_{y} \end{array}\right]\left[\begin{array}{cc} {\tilde{R}}_{11} & {\tilde{R}}_{12}\\ {\tilde{R}}_{21} & {\tilde{R}}_{22} \end{array}\right]\left[\begin{array}{c} q_{mx}\\ q_{my} \end{array}\right]\;={\tilde{R}}_{11}\tau_{x}q_{mx}+{\tilde{R}}_{21}\tau_{y}q_{mx}+{\tilde{R}}_{12}\tau_{x}q_{my}+{\tilde{R}}_{22}\tau_{y}q_{my}\;=tr\left\{\left[\begin{array}{c} {\tilde{R}}_{11}{\dot{q}}_{mx}+{\tilde{R}}_{12}{\dot{q}}_{my}\\ {\tilde{R}}_{21}{\dot{q}}_{mx}+{\tilde{R}}_{22}{\dot{q}}_{my} \end{array}\right]\left[\begin{array}{cc} \tau_{x} & \tau_{y} \end{array}\right]\right\}=tr\left\{\gamma_{R}^{-1}\tilde{R}\gamma_{R}q_{m}\tau_{0}^{T}\right\}$$

It can be seen that under the condition of asymmetric coupling coefficients, Equation (B1) still exists. Similarly the following equations can also be proved:

(B11) $$\tau_{0}\tilde{D}\left(q_{m}\left(k+1\right)-q_{m}\left(k\right)\right)=tr\left\{\gamma_{D}^{-1}\tilde{D}\gamma_{D}\left(q_{m}\left(k+1\right)-q_{m}\left(k\right)\right)\tau_{0}^{T}\right\}$$

(B12) $$\tau_{0}\tilde{\Omega }\left(q_{m}\left(k+1\right)-q_{m}\left(k\right)\right)=tr\left\{\gamma_{\Omega }^{-1}\tilde{\Omega }\gamma_{\Omega }\left(q_{m}\left(k+1\right)-q_{m}\left(k\right)\right)\tau_{0}^{T}\right\}$$

Substituting Equations (B10)–(B12) into Equation (B9):

(B13) $$\Delta V=-\gamma_{0}^{2}\Delta e^{T}\Delta e+tr\left\{\tilde{R}\left(\gamma_{R}^{-1}\Delta {\tilde{R}}^{T}-q_{m}\tau_{0}^{T}\right)+\tilde{D}\left(\gamma_{D}^{-1}\Delta {\tilde{D}}^{T}-\left(q_{m}\left(k+1\right)-q_{m}\left(k\right)\right)\tau_{0}^{T}\right)+\tilde{\Omega }\left(\gamma_{\Omega }^{-1}\Delta {\tilde{\Omega }}^{T}-\left(q_{m}\left(k+1\right)-q_{m}\left(k\right)\right)\tau_{0}^{T}\right)\right\}$$

By applying the adaptive law:

(B14) $$\tilde{R}=\gamma_{R}q_{m}\tau_{0}^{T}$$

(B15) $$\Delta \tilde{D}=\gamma_{D}\left(q_{m}\left(k+1\right)-q_{m}\left(k\right)\right)\tau_{0}^{T}$$

(B16) $$\Delta \tilde{\Omega }=\gamma_{\Omega }\left(q_{m}\left(k+1\right)-q_{m}\left(k\right)\right)\tau_{0}^{T}$$

Thus:

$$tr\left\{\tilde{R}\left(\gamma_{R}^{-1}\Delta {\tilde{R}}^{T}-q_{m}\tau_{0}^{T}\right)+\tilde{D}\left(\gamma_{D}^{-1}\Delta {\tilde{D}}^{T}-\left(q_{m}\left(k+1\right)-q_{m}\left(k\right)\right)\tau_{0}^{T}\right)+\tilde{\Omega }\left(\gamma_{\Omega }^{-1}\Delta {\tilde{\Omega }}^{T}-\left(q_{m}\left(k+1\right)-q_{m}\left(k\right)\right)\tau_{0}^{T}\right)\right\}=0$$

$\Delta V=-\gamma_{0}^{2}\Delta e^{T}\Delta e\leq 0$, and the system is proofed to be Lyapunov stable.
